# Supplementary material for: Photon-free (s)CMOS camera characterization for artifact reduction in high- and super-resolution microscopy
Source: Nat Commun. 2022 Jun 11;13:3362. doi: 10.1038/s41467-022-30907-2 (PMC9188588; doi:10.1038/s41467-022-30907-2)
Supplement: Supplementary file 1 — Supplementary Information [file 41467_2022_30907_MOESM1_ESM.pdf]

Supplementary Information

Photon-free (s)CMOS camera characterization for artifact reduction in high- and super-resolution microscopy

Robin Diekmann[1,2], Joran Deschamps[1,3], Yiming Li[1,4], Takahiro Deguchi[1], Aline Tschanz[1,5], Maurice Kahnwald[1,6], Ulf Matti[1,7], Jonas Ries[1]*

[1]Cell Biology and Biophysics Unit, European Molecular Biology Laboratory (EMBL), Heidelberg, Germany
[2]Present address: LaVision BioTec GmbH, Bielefeld, Germany
[3]Present address: Fondazione Human Technopole, Milan, Italy
[4]Present address: Department of Biomedical Engineering, Southern University of Science and Technology, Shenzhen, China
[5]Collaboration for joint PhD degree between EMBL and Heidelberg University, Faculty of Biosciences, Heidelberg, Germany
[6]Present address: Friedrich Miescher Institute for Biomedical Research, Basel, Switzerland
[7]Present address: Abberior Instruments GmbH, Göttingen, Germany
* Correspondence: jonas.ries@embl.de

Inventory of Supplementary Information

**Supplementary Figure 1**	Experiment to investigate equivalence of signal generated by either photoelectrons or thermal electrons or a mixture of both.
**Supplementary Figure 2**	Repeated photon-free characterization of the same camera shows reproducibility of the approach
**Supplementary Figure 3**	Repeated characterization of the same camera three years apart
**Supplementary Figure 4**	Comparison of ACCeNT calculation and direct measurement on single pixel level
**Supplementary Figure 5**	Characteristics of and STORM example images recorded by two uncooled, industry-grade CMOS cameras
**Supplementary Figure 6**	Nonlinear effects observed for a cooled, scientific-grade CMOS camera
**Supplementary Figure 7**	Uncorrected CMOS pixel-to-pixel variations affect localization accuracy both laterally and axially
**Supplementary Figure 8**	Application of CMOS specific fitting benefits from explicit correction of thermal effects
**Supplementary Note 1**	Maximum likelihood localization in the presence of sCMOS specific noise
**Supplementary Note 2**	Software Manual
**Supplementary Video 1**	3D reconstruction of the four nuclear pore complexes
**Supplementary Video 2**	Time-lapse live-cell TIRF data of GFP-tagged AP2

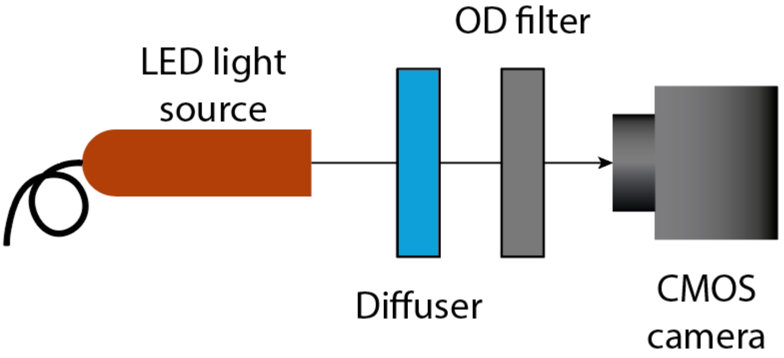

Supplementary Figure 1: Experiment to investigate equivalence of signal generated by either photoelectrons or thermal electrons or a mixture of both

An LED lamp was directed via a diffuser and an OD filter towards the sensor of an uncooled, industry-grade camera. Combinations of different brightness levels at the camera and different exposure times were adjusted to yield the same average detected signal by the camera and 8000 frames were recorded. The following four configurations are plotted in Figure 1a for one specific pixel: 1 ms exposure time and no light to the camera (intended measurement of read noise), 1 ms exposure time and light to the camera (intended measurement of read noise and photoelectron induced noise), 100 ms exposure time and less light to the camera (intended measurement of read noise, photoelectron induced noise and thermal noise), 500 ms exposure time and no light to the camera (measurement of read noise and thermal noise). Note that we selected one pixel with similar mean signal in all configurations as the dark current strongly varies between pixels (Supplementary Figure 2).

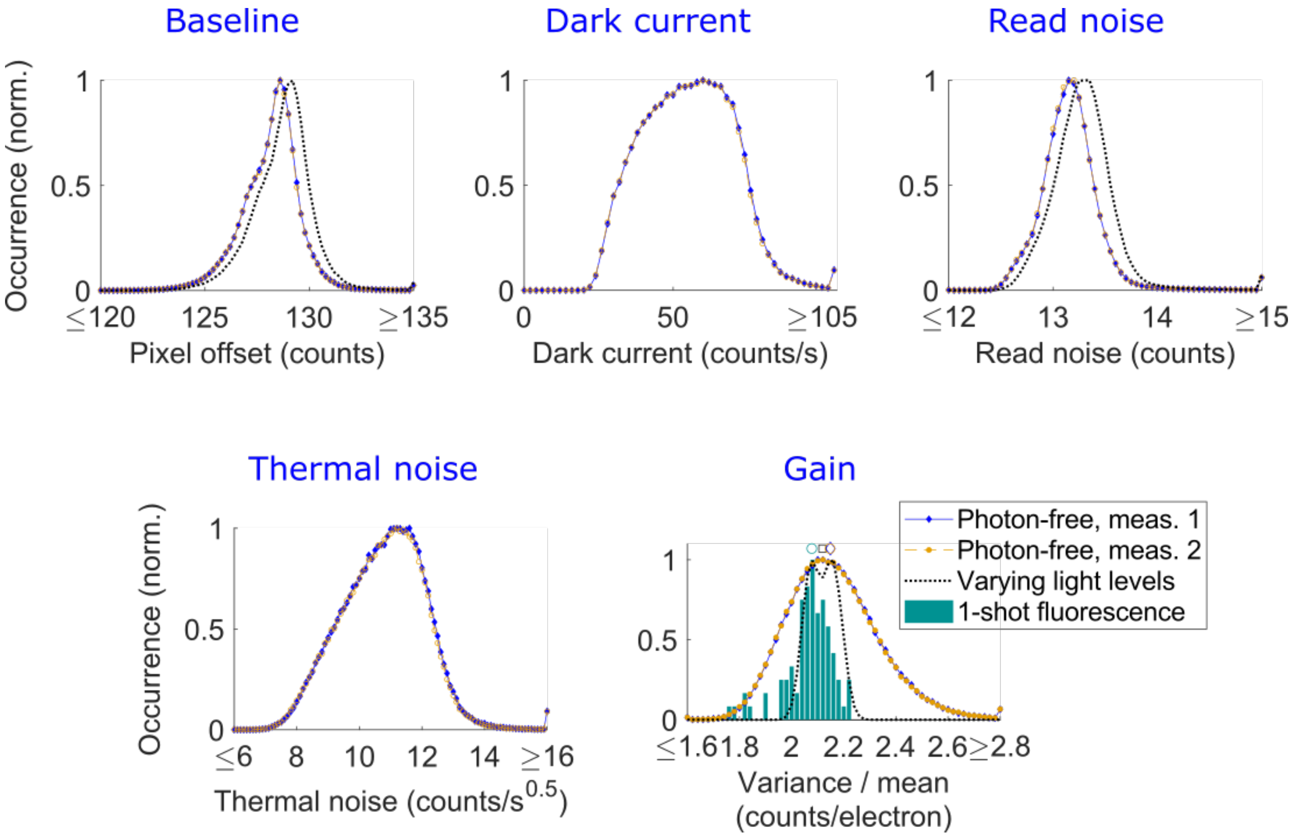

Supplementary Figure 2: Repeated photon-free characterization of the same camera shows reproducibility of the approach

Comparison of repeated photon-free characterizations of the same camera show high similarity (blue and orange curves). The photon-free characterization measurements were performed on the same day. For comparison: Histograms of pixel values obtained by photon-free characterization (blue curve and orange curve) and traditional characterization of using varying light levels (black dashed curve). Distribution of the gain determined via different approaches (pixelwise histogram for the photon-free and varying light levels approaches, histogram of outcomes from multiple determinations of the mean gain from the 1-shot approach). Symbols above the curves indicate the medians (blue diamond and orange circle for photon-free approach, square for vary light levels approach, circle for 1-shot fluorescence).

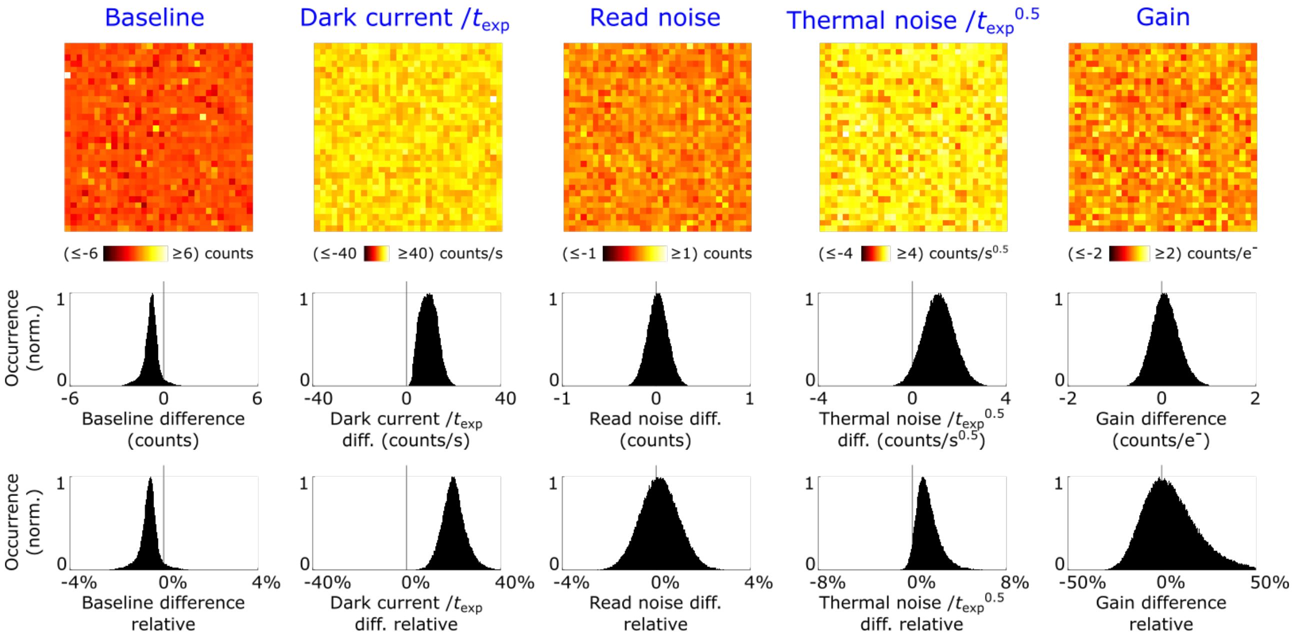

Supplementary Figure 3: Repeated characterization of the same camera three years apart
Comparison of repeated photon-free characterizations of the same camera three years apart. The maps show the difference on a single pixel level for a region of 32x32 pixels. The histograms show the difference for all pixels of the central 512x512 pixels of the industry-grade camera in absolute values (middle row) and relative values (bottom row). The statistics of the changed parameters over three years are the following (mean ± standard deviation). Baseline difference: (-0.81 ± 0.76) counts absolute, (-0.62 ± 0.46) % relative; Dark current difference: (9.4 ± 3.8) counts/s absolute, (19.9 ± 5.3) % relative; Read Noise difference: (0.02 ± 0.13) counts absolute, (0.14 ± 0.94) % relative; Thermal Noise difference: (1.13 ± 0.87) counts/s$^{0.5}$ absolute, (1.3 ± 1.2) % relative. Gain difference: (0.08 ± 0.30) counts/electron absolute, (5 ± 14) % relative. The gain median over all pixels changed by 0.083 counts/electron absolute and 4 % relative. We speculate that the differences in dark current and thermal noise can be attributed to a different mounting of the camera acting as a heat sink, leading to slightly different effective operating temperatures of the camera.

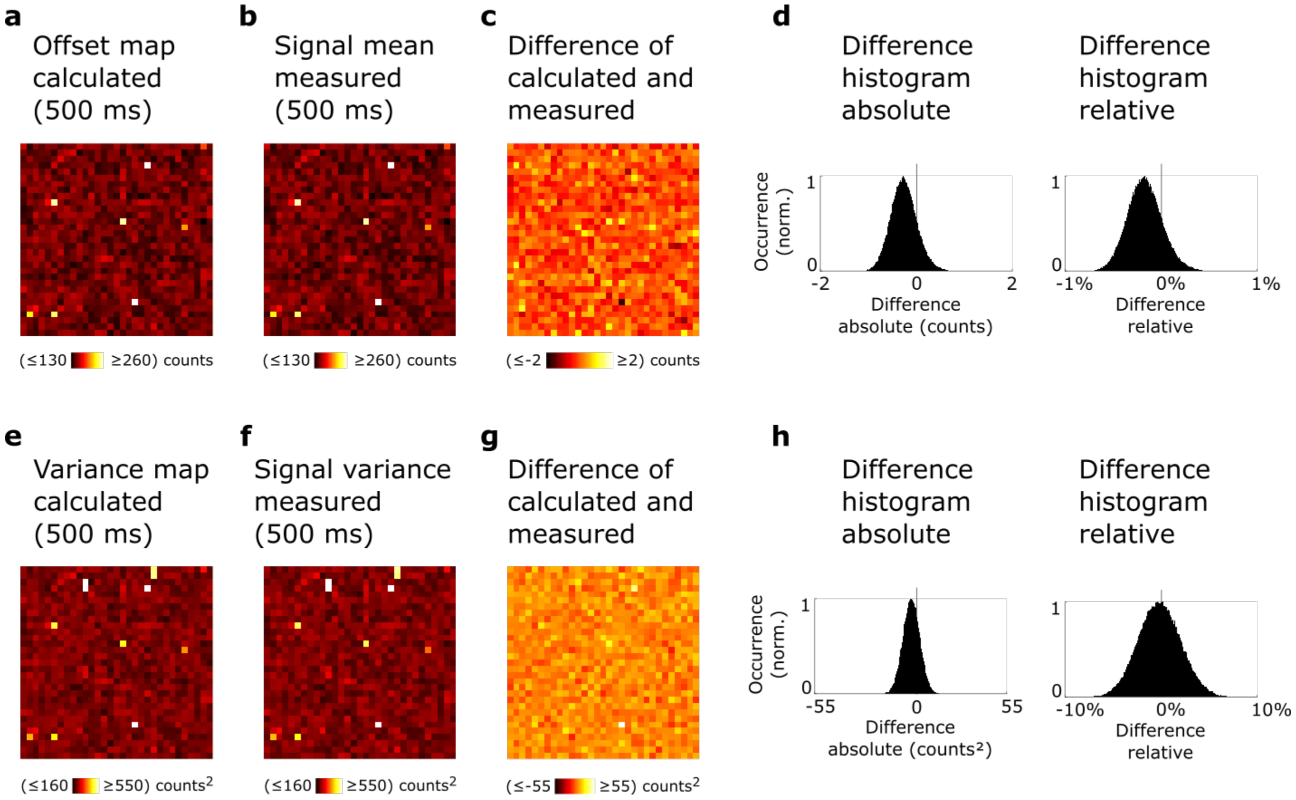

Supplementary Figure 4: Comparison of ACCeNT calculation and direct measurement on single pixel level

An industry-grade CMOS camera was characterized using photon-free measurements at the exposure time points of 1 ms, 100 ms, 200 ms, 300 ms and 400 ms. Following the workflow depicted in Figure 1d, the offset (**a**) and variance (**e**) were calculated by extrapolation to an exposure time of 500 ms. An additional stack of dark frames at 500 ms exposure time was recorded and mean (**b**) and variance (**f**) were calculated on a single pixel level. **c**,**g** show the corresponding difference maps for the calculation and direct measurement for the same region of 32x32 pixels. **d**,**h** show the histograms in the absolute and relative differences over all central 512x512 pixels of the sensor. The statistics of the differences are the following (mean ± standard deviation). Offset difference: (-0.26 ± 0.29) counts absolute, (-0.17 ± 0.19) % relative; Variance difference: (-0.4 ± 5.0) counts² absolute, (-0.1 ± 2.2) % relative.

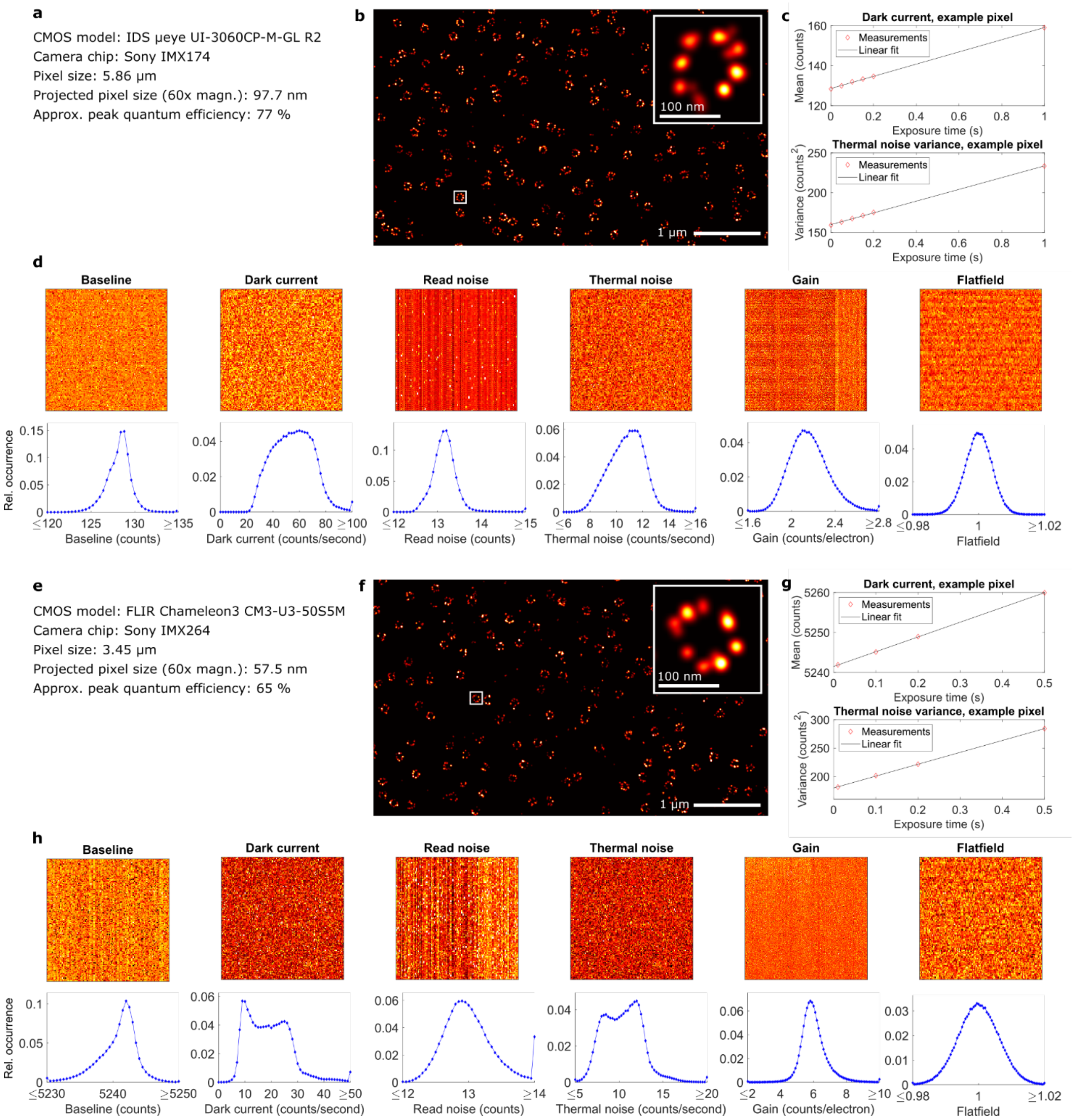

Supplementary Figure 5: Characteristics of and STORM example images recorded by two uncooled, industry-grade CMOS cameras

a,e, Camera parameters, **b,f,** example STORM reconstructions, **c,g,** example data for exposure-time dependent signal and noise and **d,h,** measured camera characteristics for two different industry-grade CMOS cameras.

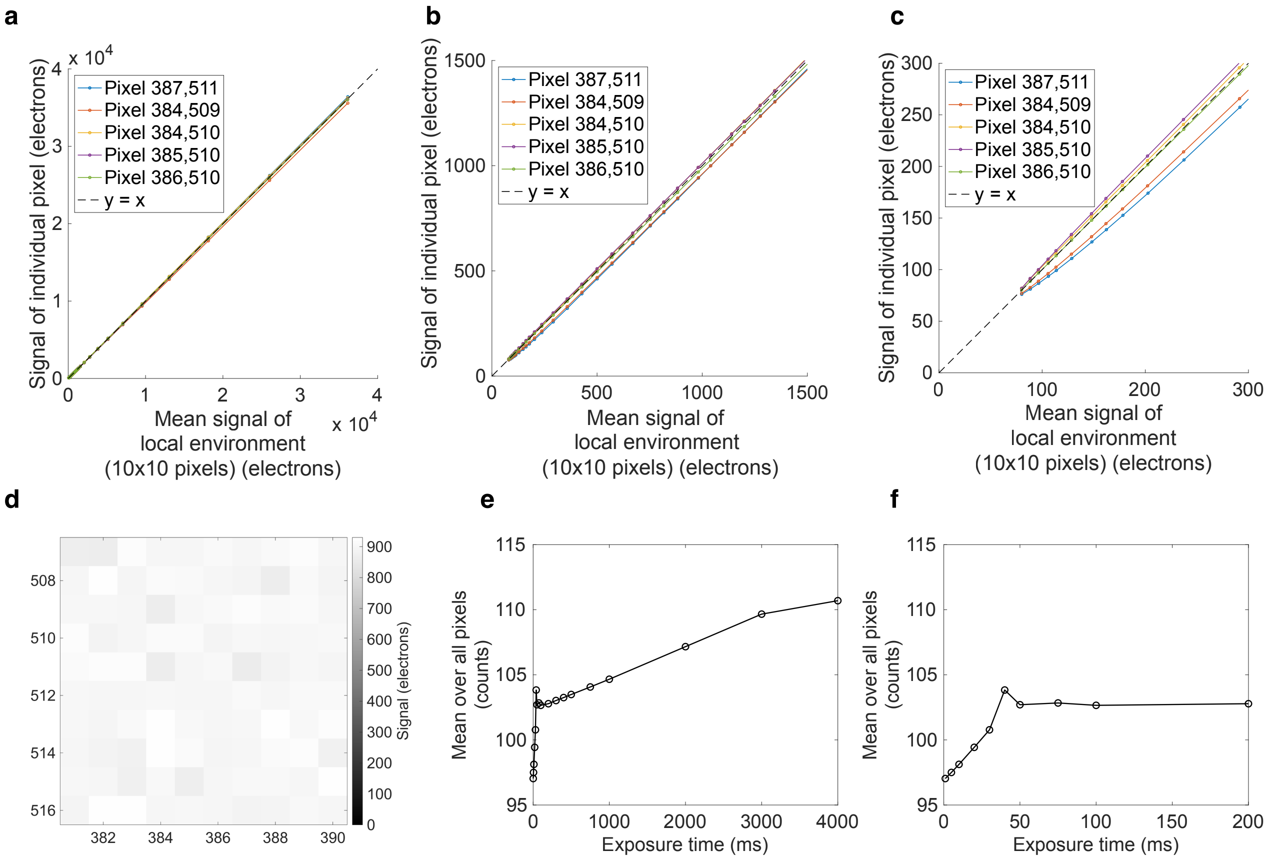

Supplementary Figure 6: Nonlinear effects observed for a cooled, scientific-grade CMOS camera

a-**c**, We applied the traditional characterization routine of varying light levels (ensuring a spatially mostly homogeneous illumination) to our cooled, scientific-grade CMOS camera (Edge 4.2 bi, PCO) and plot the mean signal per illumination condition as a function of the mean signal of the local 10x10 pixel neighborhood for selected pixels. **a** shows 0 % to 72 % of the dynamic range of the camera, **b** shows a zoom into 0 % to 3 % of the dynamic range and c shows a zoom into 0 % to 0.06 %. While the light response of the camera is mostly linear over a large part of the dynamic range, the zoom in (**c**) reveals nonlinearities for some pixels particularly for about the first 200 (photo)electrons above the offset. For the DNA-PAINT experiments presented in this work, the mean background per pixel was 301 photons (and the fluorescence signal above the background was much higher), so we operate the camera in the linear regime. **d** shows the mean signal for a part of the camera. **e**,**f**, We recorded dark images at different exposure times and plot the mean signal of all pixels as a function of the exposure time. **e** shows the plot for 0 ms to 4,000 ms and **f** shows a zoom into 0 ms to 200 ms. The plot reveals three different regimes of dark current for the camera: The dark current appears proportional to the exposure time for 0 ms to 30 ms (corresponding to high to medium framerates often used in STORM and PALM imaging), some transitioning behavior for exposure times from 40 ms to 200 ms, and again appears proportional to the exposure time for 200 ms to 3000 ms. For the experiments presented here, we characterized the camera via the photon-free approach for exposure times from 350 ms to 3000 ms and operate the camera at 500 ms for the DNA-PAINT imaging, so the characterization as well as the experiments are performed in a linear regime.

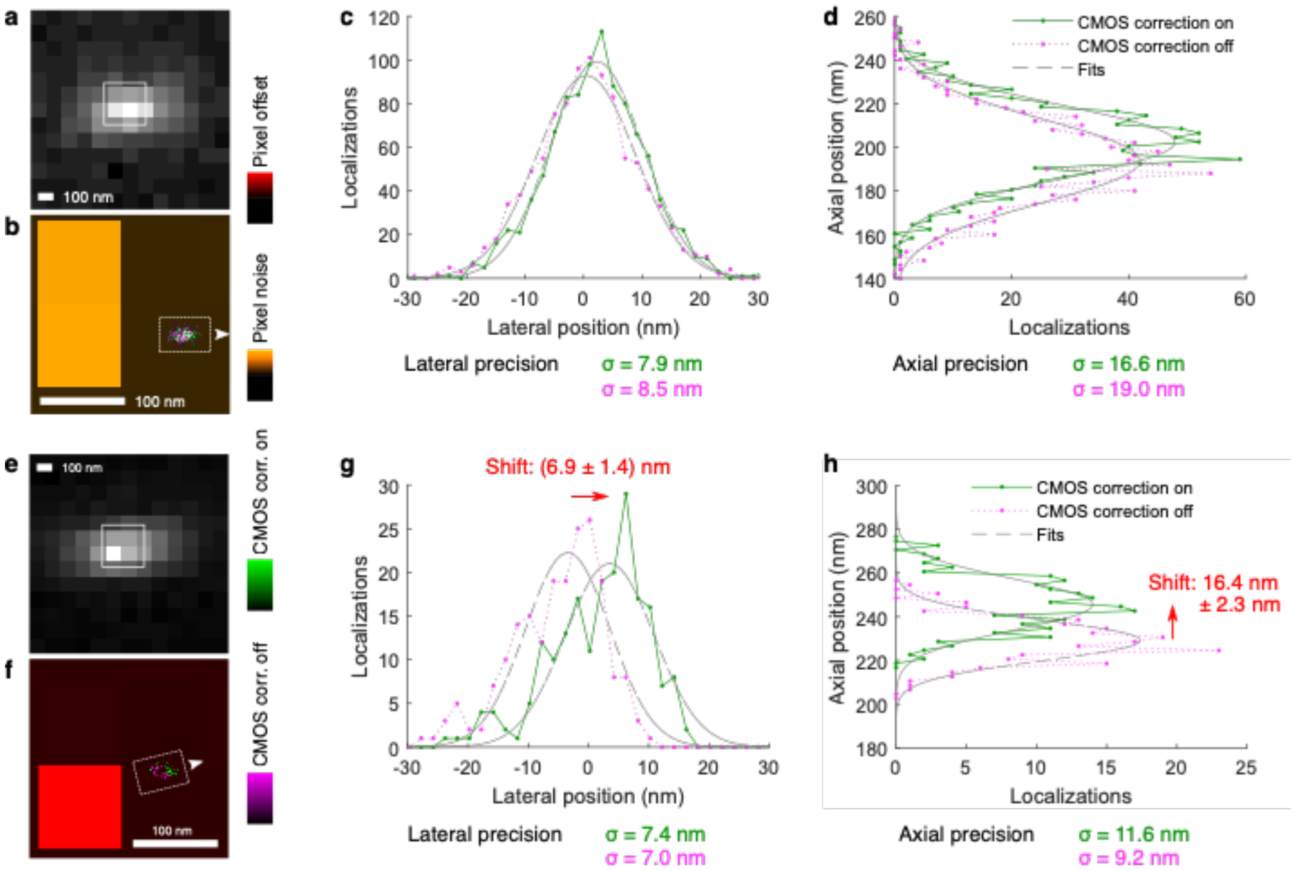

Supplementary Figure 7: Uncorrected CMOS pixel-to-pixel variations affect localization accuracy both laterally and axially

a, A fluorescent bead was positioned such that its diffraction limited image was captured close to two pixels of increased noise. **b** shows a zoom into the region indicated in **a** including the pixel noise characteristics and the repeated localizations via astigmatism-based 3D PSF fitting. Magenta dots show the localizations when not using an (s)CMOS-specific fitter that takes care of individual pixel characteristics and green dots show the localizations when using an (s)CMOS-specific fitter that explicitly considers individual pixel characteristics including noise. Gaussian fits to line profiles of the localization distribution inside the boxed region indicated in **b** show that the localization precision (i.e. the standard deviation of the localizations) is increased when not using the (s)CMOS-specific fitter both laterally (**c**) and axially (**d**).

e,**f**, Repeating the same experiment, but capturing the image of a bead in proximity to a pixel of high offset resulting from high dark current. In this case, the mean localized position is significantly shifted both laterally (**g**) and axially (**h**). The axial shift occurs because the axial position is being estimated from the shape of the PSF which becomes apparently distorted by the pixel of increased brightness when not applying a (s)CMOS-specific fitter that explicitly considers individual pixel characteristics including offset.

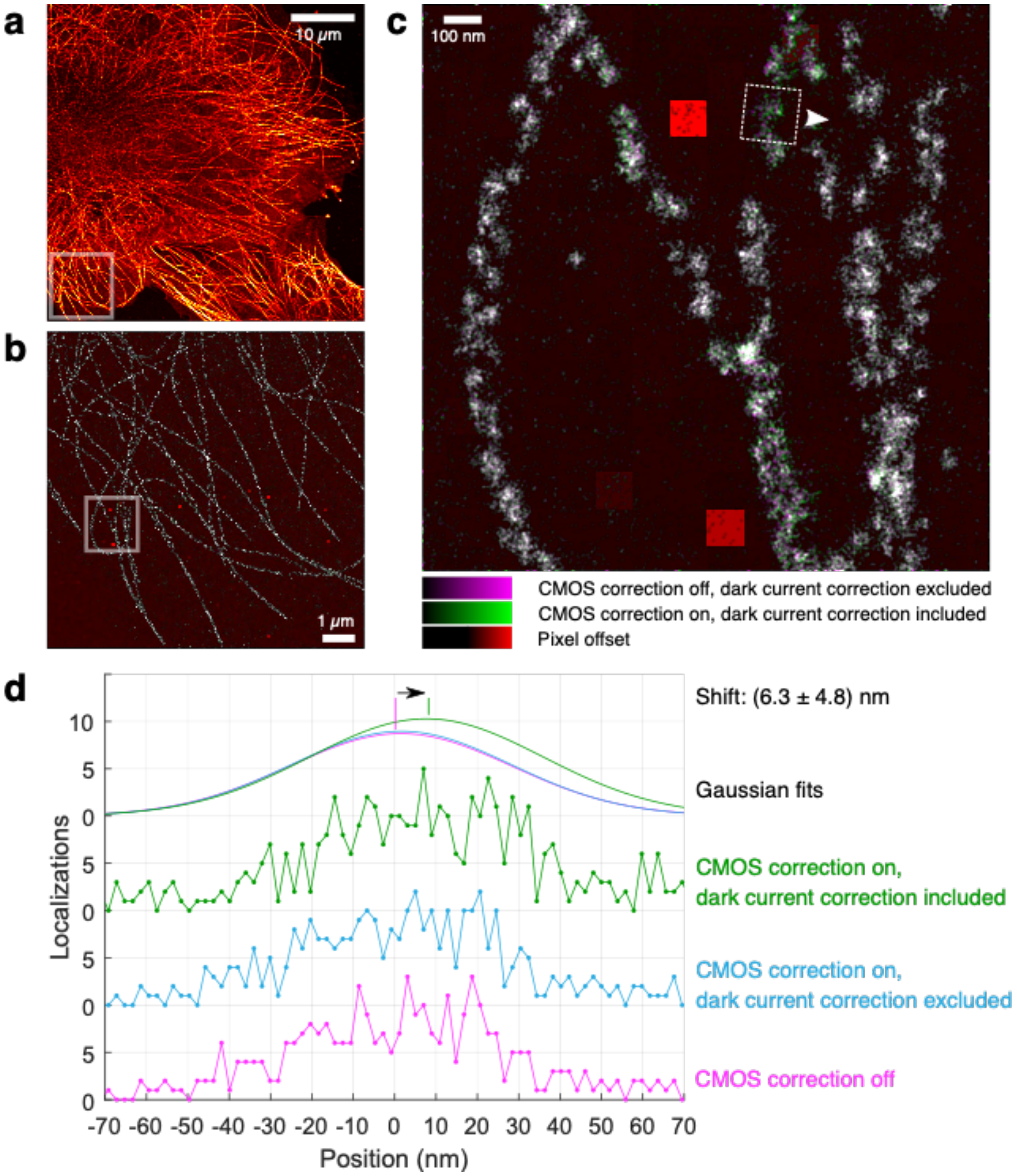

Supplementary Figure 8: Application of CMOS specific fitting benefits from explicit correction of thermal effects

a, DNA-PAINT image of microtubules in a U2OS cell recorded using an uncooled, industry-grade CMOS camera. **b** shows a zoom into the region indicated by the box in **a** and **c** shows a zoom into the region indicated by the box in **b**. Localizations fitted without CMOS correction are displayed in magenta while the same localizations fitted with CMOS correction including thermal effects are displayed in green. The pixel offset, of which variations mainly stem from dark current, is displayed in red for the underlying pixel grid of the camera. **d**, line profiles according to the region indicated in **c** and Gaussian fits to determine the center position of the structure. The center becomes shifted by about 6 nm when not applying CMOS-specific fitting and, more notably, this shift persists even when the CMOS correction is applied in principle, but thermal effects are neglected.

Supplementary Note 1: Maximum likelihood localization in the presence of sCMOS specific noise

Huang *et al.* introduced a clever way to account for pixel-specific read-noise in maximum likelihood estimation (MLE) based fitting[1]. By approximating the normal distributed readout noise (v_k in the unit of electrons square, k denotes the pixel indices) with a Poisson distribution, one can expect the sum of the measured photoelectrons D_k in each pixel and the pixel-dependent readout noise v_k to approximate a Poisson distribution with the mean of $u_k + v_k$:

$$P(x = (D_k + v_k)|u_k, v_k) = \frac{e^{-(u_k+v_k)}(u_k + v_k)^x}{\Gamma(x + 1)}$$

Here, u_k is the expected photoelectrons in pixel k calculated from the PSF model. Therefore, the conventional MLE fit for Poisson distribution can be easily applied for the pixel-dependent (s)CMOS data by adding the readout noise v_k to the measured photoelectron D_k as the observed data and update the model function u_k with $u_k + v_k$ as the expected value. Since the exposure time-dependent thermal noise and dark current both follow the Poisson distribution and they are effectively added in the finally detected electrons, we can assume that $D_k + v_k$ will approximate a Poisson distribution with the variance of $u_k + v_k + T_k^2 * t$. Here, T_k^2 denotes the variance introduced from thermal noise in the k_{th} pixel per time (electrons/second). t is the camera exposure time. Compared to Huang's method considering only the effect of pixel dependent readout noise, we further take account of the effect of pixel and exposure time dependent dark current and thermal noise and incorporate them into our MLE fitting model.

Supplementary Note 2: Software Manual

Introduction

The complete ACCeNT workflow for camera characterization and SMLM fitting can be performed in two different ways:

- *EITHER* using the Micro-Manager 2 ACCeNT plugin for acquisition and simultaneous ACCeNT camera map computation ("Micro-Manager 2 calibration") followed by SMAP SMLM fitting ("Single-molecule localization using SMAP");
- *OR* using the Micro-Manager 1.4 ACCeNT script for acquisition followed by using the Fiji plugin for ACCeNT camera map computation ("Micro-Manager 1.4 and Fiji calibration") followed by using SMAP for SMLM fitting ("Single-molecule localization using SMAP").

Since all implementations are either available as plugins or scripts for FIJI or Micro-Manager, no specific installation of ACCeNT is necessary.

As an alternative to the complete workflow, users can choose to perform only certain parts of the pipeline. For instance, the Micro-Manager 2 plugin allows to perform acquisition and computation independently, or the Fiji plugin can be used for computation of appropriately acquired raw camera data from other software.

To test our pipeline on provided example data, start at point 2. of the "Micro Manager 1.4 and Fiji calibration" workflow below. This includes using Fiji for ACCeNT camera calibration and using SMAP for fitting of 3D SMLM data. The example data corresponds to the 3D STORM data of nuclear pore complexes shown in Fig. 3. The example data has been recorded using an uncooled, industry-grade CMOS camera (IDS UI-3060CP-M-GL Rev.2, Sensor Sony IMX174) and can be found here: https://rieslab.de/#accent.

We successfully tested ACCeNT with the following cameras:

- UI-3060CP-M-GL Rev.2, IDS
- Edge 4.2bi, PCO
- DCC1545M, Thorlabs
- Chameleon3 CM3-U3-50S5M, FLIR
- Chameleon3 CM3-U3-31S4M-CS, FLIR
- Prime BSI, Photometrics

We did not manage to run ACCeNT with the Hamamatsu Orca Flash4.0v2 because of an apparent dark current correction implemented by the manufacturer.

The software of ACCeNT is licensed under GNU GPL v3.0.

Micro-Manager 2 calibration

1. Installation
    a. Download the latest version of Micro-Manager 2 from:
        - https://micro-manager.org/wiki/Download_Micro-Manager_Latest_Release
    b. Download the latest release of the ACCeNT Micro-Manager 2 plugin:
        - https://github.com/ries-lab/Accent/releases
    c. Place the downloaded .jar in the "mmplugins" folder of your Micro-Manager 2 installation folder.
2. Start Micro-Manager 2 using a hardware configuration that includes your camera. Refer to the Micro-Manager wiki or the Image.sc forum for any trouble regarding hardware.
    a. ACCeNT can be started from the plugins menu, under "Acquisition Tools". The plugin consists of three steps: acquisition, processing and maps generation. All steps can be carried out together or separately.

3. Set ACCeNT parameters:
    a. Using the "..." button, select a folder in which the images will be saved.
    b. Select the name you want to give the images, the number of frames per exposure and the exposures. We advise a minimum of 15000 frames and 3 exposure times.
    c. By clicking on "Options", you can select further acquisition options:
        - Pre-run (min): pre-acquisition run time to thermally equilibrate the camera. This is particularly necessary in the case of uncooled cameras. The total run time is an approximation as overhead exists and is camera dependent.
        - Save frames as: allows saving the frames as tiff stacks or individual images.
        - Process data: process the calibration live (in parallel) or wait for the user to start it. If the acquisition is faster than the processing, the plugin will run into a buffer overflow and crash. In such a case, run your calibration with the "separately" option.
        - Roi: sets the roi on which to perform the calibration. Roi set by Micro-Manager are ignored. If all fields are 0, then the calibration is performed on the full chip.

    Save the options to validate them.
4. Click on "Run" to start the acquisition. If the "in parallel" processing options was chosen, the processing also starts. The acquisition step saves the images, as well as a JSON representation of the roi (roi.roi file).
5. Once the acquisition is done, the folder and roi fields of the processing panel are updated.
        - If the processing was chosen to be in parallel to the acquisition, then it finishes soon after the last image is acquired.
        - If the processing is done separately, click on "Process".

    The processing step can be performed later by just loading the folder containing the images using the "..." button. The plugin automatically detects the roi.roi file and updates the fields. If no roi file is present, input the roi x, y, width and height manually.
6. The processing steps creates a calibration file (results.calb), an average and variance map for each exposure, as well as images of the various estimates present in the calibration.
7. The map generation step is automatically performed after each processing step. To run a new generation step, you can load a calibration file using the "..." button. Input the desired exposures in the exposure field and click on "Generate". The map generation step generates the average and variance maps for the required exposures.

The calibration file (results.calb) can then be used with SMAP.

Micro-Manager 1.4 and Fiji calibration

In this pipeline, we make use of Micro-Manager 1.4 to generate the data. In principle the Fiji plugin can be used regardless of the way the raw data was generated. The only conditions are:

- The images are tiff (stacks or individual images)
- Each stack's name contains "XXXms", where XXX is the corresponding exposure time, and all stacks are in the same folder. **Alternatively**, each exposure (stack or individual images) can be in a folder with name containing "XXXms". All exposure folders must be grouped in one folder.

1. Acquire camera frames with Micro-Manager 1.4.
    a. Download the latest version of Micro-Manager 1.4 from:
    https://micro-manager.org/wiki/Download_Micro-Manager_Latest_Release
    b. Start Micro-Manager 1.4 with a hardware configuration containing your camera.
    c. In "Tools", open the "Scripting panel".

d. Then load "accent-acquisition_script.bsh" from the Github repository:

   https://github.com/ries-lab/Accent/blob/master/accent-mm1/accent-acquisition_script.bsh

e. In the script, modify the parameters "path", "exposures" and "numFrames". Additionally, change the roi on line 12. The parameters of the setRoi method are in order x0, y0, width and height; with x0 and y0 defining the top-left corner of the roi. Note that certain cameras do not support this function. In such case, comment out (using "//") line 12 and set the roi manually in Micro-Manager (refer to Micro-Manager user's guide)

f. Run the script to acquire the images. This can take some time, watch the console for progress and wait for the acquisition to complete.

2. **Start here to test ACCeNT on provided example data.** The example data can be downloaded at https://rieslab.de/#accent. The zip-archive for the example data contains raw data from the camera calibration, 3D stacks of beads for the point spread function (PSF) characterization and raw STORM data corresponding to Figure 3k-m.

3. Install Fiji

   a. Download Fiji:

      https://imagej.net/Fiji/Downloads
      Please note that the ACCeNT plugin uses features that have been added recently to Fiji. If you encounter errors, make sure to use the latest version of Fiji.

   b. Download the latest release of the ACCeNT Fiji plugin:

      https://github.com/ries-lab/Accent/releases

   c. Place the downloaded accent-fiji-1.0.jar in the "plugins" folder of your Fiji installation folder.

4. Start Fiji and the ACCeNT plugin from the plugins menu.

5. Set ACCeNT parameters:

   a. Detect the images by selecting the folder in which they are contained using the "..." button. They should appear in the table with the correct exposure time in the second column.

      - The example raw ACCeNT data is located in the folder "ACCENT_raw"

   b. Set the roi parameters x (X0), and y (Y0), e.g. used in the Micro-Manager 1.4 acquisition under point 5.

      - For the example data, keep the default values X0 = 0 and Y0 = 0.

6. Click on "Process" and wait.

   Processing of the example data takes about 20 minutes (Windows 64-bit operating system, i5-4690K CPU @ 4x3.5GHz, 32 GB memory).

7. Output files:

   a. The processing steps creates a calibration file (results.calb) to be used with SMAP, as well as different camera maps (in units of ADU counts unless stated otherwise):

   b. The mean pixel values for each exposure time are saved as Avg_XXXms.tiff, where XXX denotes the exposure time in ms.

   c. The variance pixel values for each exposure time are saved as Var_XXXms.tif, where XXX denotes the exposure time in ms.

   d. The computed baseline is saved as Baseline.tiff.

   e. The computed dark current per 1 second is saved as DC-per_sec.tiff.

   f. The square of the computed read noise (in units of ADU counts squared) is saved as RN_sq.tiff.

g. The square of the thermal noise per 1 second (in units of ADU counts squared) is saved as TN_sq_per_sec.tiff.
h. The computed gain (in units of ADU counts per electron) is saved as Gain.tiff.
i. The computed offset maps for arbitrary exposure times (default values are 15 ms, 20 ms, 30 ms, 50 ms and 100 ms) are saved as generated_Avg_XXXms.tiff, where XXX denotes the exposure time in ms.
j. The computed variance maps for arbitrary exposure times (default values are 15 ms, 20 ms, 30 ms, 50 ms and 100 ms) are saved as generated_Var_XXXms.tiff, where XXX denotes the exposure time in ms.
k. The R_sq_YYY.tiff files show the R^2 values for inspection of the quality of the fits used to compute baseline (R_sq_avg.tiff), read noise squared (R_sq_var.tiff) and gain (R_sq_gain.tiff).
    - You can inspect the generated camera maps of the example data using Fiji. For instance, open the "DC_per_sec.tiff" in Fiji. While the mean dark current is 54 counts/s (using Analyze -> Measure; set mean via Results -> Set Measurement), you will find that pixel (256,289) features significantly higher dark current of 1429 counts/s (placing the cursor over the pixel reading the value from the main Fiji window). Visual inspection of the image shows other pixels of pronounced dark current, e.g. (220,315), (289,284) and (266,247). To find the dark current in units of electrons/s, open the "Gain.tiff" file and measure the median gain value (using Analyze -> Measure; set median via Results -> Set Measurement). For the example data, the gain is 2.16 counts/electron. Hence, the mean dark current is (54 counts/s) / (2.16 counts/electron) = 25 electrons/s.
    - To inspect the read noise, open the "RN_sq.tiff" and calculate its square root (using Process -> Math -> Square Root). The mean read noise is 13.2 counts, corresponding to (13.2 counts) / (2.16 counts/electron) = 6.1 electrons.

8. Though not needed for fitting of single molecule localization data in SMAP, ACCeNT can create exposure time specific camera maps. These can for instance be used to explore camera characteristics, find particularly bad pixels, or as input for software other than SMAP. The map generation step is automatically performed after each processing step. To run a new generation step, you can load a calibration file using the "..." button. Input the desired exposures in the exposure field and click on "Generate". The map generation step generates the average and variance maps for the required exposures.
    - For the example data, type "999" into the field "Exposures (ms):" and click "Generate". The generated camera maps for offset and variance corresponding to 999 ms exposure time will be saved to the folder "ACCeNT_raw". To check the consistency with the measurement, open both the "Avg_999ms.tiff" (the mean pixel values directly from the measured data) and the "generated_Avg_999ms.tiff" (the computed offset values for this exposure time) files in Fiji. Compute the difference (using Process -> Image calculator...; Image 1: Avg_999ms.tiff, Operation: Subtract, Image 2: generated_Avg_999ms.tiff, check Create new window, check 32-bit (float) result). The mean difference between the measured and computed maps is –0.042 counts corresponding to (-0.042 counts) / (2.16 counts/electron) = -0.019 electrons.

Single-molecule localization using SMAP

1. Install SMAP from: https://github.com/jries/SMAP. The version from time of publication is available at: https://github.com/jries/SMAP/tree/v220315.

   following the instructions. Alternatively, a stand-alone version for PC and Mac can be downloaded at: https://rieslab.de/#software (version 220315 or newer).

   Please follow the installation instructions provided with the executables.

Familiarize yourself with SMAP by consulting the documentation, using example data downloaded at https://rieslab.de/#accent. Make sure to install Micro-Manager **V1.4** (https://micro-manager.org) and select its path in the Preferences menu.

2. As described in the user guide (SMAP_UserGuide.pdf (embl.de), page 6), add your camera to the camera manager.

   For the example data, the camera is already registered in the camera manager.

3. You need to specify in SMAP the location of the calibration file: In the 'correctionfile' field, select the camera calibration file, for the example the results.calb file. Save the changes in the camera manager.

4. For fitting of 3D SMLM data, perform the 3D calibration as described in the SMAP user guide (https://www.embl.de/download/ries/Documentation/SMAP_UserGuide.pdf, page 9 "experimental PSF model"). For the provided example data, you can EITHER skip this step and use the file:

   "60xOil_sampleHolderInv__CC0.140_1_MMStack.ome_3dcal.mat"

   from the "PSF_raw" folder in the second next step, OR perform the 3D PSF calibration yourself, based on 3D stacks of 100 nm Tetraspeck beads:

   - Open "Plugins -> Analyze -> sr3D -> calibrate3DsplinePSF"
   - On the new window, click "Run"
   - On the new window, click "Select camera files"
   - On the new window, click "add dir"
   - Navigate to the "PSF_raw" folder and mark all folders
   - Click "Open"
   - In the previous window, click "Done"
   - In the previous window, click "Calculate bead calibration" and wait. Processing of the example data takes about 2 minutes (Windows 64-bit operating system, i5-4690K CPU @ 4x3.5GHz, 32 GB memory, GeForce GTX 970 GPU).

5. In the SMAP main window select the 'Localize' tab and the 'Input Image' subtab to load the raw SMLM data via "load images".

6. For the example data, open the first Tiff-file in the "SMLM_raw folder". Click "set Cam Parameters" to validate that the camera has been recognized. Do not change any values and click "OK". The example data corresponds to the 3D STORM data of nuclear pore complexes shown in Fig. 3k-m. The example data has been recorded using an uncooled, industry-grade CMOS camera (IDS UI-3060CP-M-GL Rev.2, Sensor Sony IMX174). It contains 19000 frames. The recording was manually stopped after bleaching of most fluorophores. Check 'correct offset'.

7. In the SMAP main window, change to the 'Fitter' subtab, select 'Spline' as the fitting model via the drop-down menu, click "Load 3D cal" and load the 3D PSF calibration file.

   For the example data, load the file:

   "60xOil_sampleHolderInv__CC0.140_1_MMStack.ome_3dcal.mat".

8. Check "sCMOS correction".

9. Check "RI mismatch"

10. Click "Preview" to test fitting.

11. If successful (i.e. you get the message "preview done"), press "Localize" to start the localization process.

    For the provided example data, this takes about 6 minutes (Windows 64-bit operating system, i5-4690K CPU @ 4x3.5GHz, 32 GB memory, GeForce GTX 970 GPU). The file containing the localizations is saved as "SMLM_raw_sml.mat" in the main folder.

12. You can repeat the fitting process by unchecking the “sCMOS” check box in the “Fitter” tab, unchecking the “Correct offset” checkbox in the “Input Image” tab and compare the results.

    For the provided example data, go to the “Input Image” tab and unclick “Correct offset”, go to the “Fitter” tab and unclick “sCMOS”, click “Localize” and wait. This will take another about 6 minutes.

    - In the main window, click “Load” and navigate to the main folder of the example data. Mark both “SMLM_raw_sml.mat” (the fitting results using the CMOS fitter with the ACCeNT calibration) and “SMLM_raw_2_sml.mat” (the fitting result not using the CMOS fitter). Click “Open”. If you have not performed the fitting step yourself, you can alternatively load the provided localization files “provided_SMLM_CMOS_fitter_sml.mat” and “provided_SMLM_no_correction_sml.mat”.
    - Change to the “Render” tab.
    - In the drop-down menu, select “ SMLM_raw_sml”
    - Change “LUT:” to “green” via the drop-down menu.
    - Next to “quantile”, change the value to “–3.0”
    - Set the localization filters on the right to the following intervals (lower bound is left value, upper bound is right value):
        - locprec: [0, 30]
        - z: [-200, 300]
        - frame: [600, Inf]
    - Next to “Layer1”, click “+”.
    - In the drop-down menu for the new “Layer2”, select “ SMLM_raw_2_sml”
    - Click the button “frame” to re-activate the frame filter.
    - Click “Inv” next to “LUT:”.
    - Click “Render”.
    - In the “format” region of the main window, click “Reset”.
    - To perform drift correction, change to the “Process” tab, check “Correct z-drift” and click “Run.” Drift correction will take about 2 minutes.
    - Go back to the “Render” tab and click “Render”.
    - In the “format” region of the main window, change the value of Pixrec (nm) to “3” and press enter.

    You can now inspect the reconstructed STORM image. Move around by right-clicking the region you want to bring to the center of the ROI. Data that has been fitted with the CMOS fitter is shown in green, data that has been fitted without the CMOS fitter is shown in magenta. Accordingly, regions where localizations from both fitting processes coincide well are displayed greyish.

    To visualize the reconstructed STORM image in 3D, in the “layers” part of the main window, uncheck the box of “2”. Go to “Layer1”, change “Colormode:” to “z”, change “LUT:” to “jet”, change the values next to “c range” to –100. In the “format” region of the main window, change the value of “Pixrec (nm)” to 10, press enter and click “Render”. The z-coordinate of the dataset that has been fitted using the CMOS-specific fitter is now color-coded. Move around by right-clicking the region you want to bring to the center of the ROI.
