# Supplementary material for: Photon-free (s)CMOS camera characterization for artifact reduction in high- and super-resolution microscopy
Source: Nat Commun. 2022 Jun 11;13:3362. doi: 10.1038/s41467-022-30907-2 (PMC9188588; doi:10.1038/s41467-022-30907-2)
Supplement: Supplementary file 3 — Description of Additional Supplementary Files [file 41467_2022_30907_MOESM3_ESM.docx]

**Description of Additional Supplementary Files**

**Supplementary Video 1:** 3D reconstruction of the four nuclear pore complexes shown in Figure 2m.

**Supplementary Video 2:** Time-lapse live-cell TIRF data of GFP-tagged AP2 in U373 cells recorded using an uncooled, industry-grade CMOS camera. Left: Unprocessed data directly from the camera, Center: NCS-processed data after ACCeNT-calibration of the camera, Right: ACsN-processed data after ACCeNT-calibration of the camera. Note that for the ACsN algorithm, the parameter for the projected pixel width was chosen as 108 nm (instead of 98 nm which corresponds to the physically correct value) since the correct value led to a considerable loss in resolution.
