# Supplementary material for: Photon-free (s)CMOS camera characterization for artifact reduction in high- and super-resolution microscopy
Source: Nat Commun. 2022 Jun 11;13:3362. doi: 10.1038/s41467-022-30907-2 (PMC9188588; doi:10.1038/s41467-022-30907-2)
Supplement: Supplementary file 6 — Reporting Summary [file 41467_2022_30907_MOESM6_ESM.pdf]

nature portfolio

Corresponding author(s): Jonas Ries

Last updated by author(s): March 28th 2022

Reporting Summary

Nature Portfolio wishes to improve the reproducibility of the work that we publish. This form provides structure for consistency and transparency in reporting. For further information on Nature Portfolio policies, see our Editorial Policies and the Editorial Policy Checklist.

Statistics

For all statistical analyses, confirm that the following items are present in the figure legend, table legend, main text, or Methods section.

n/a	Confirmed	
☐	☒	The exact sample size (*n*) for each experimental group/condition, given as a discrete number and unit of measurement
☐	☒	A statement on whether measurements were taken from distinct samples or whether the same sample was measured repeatedly
☒	☐	The statistical test(s) used AND whether they are one- or two-sided<br>*Only common tests should be described solely by name; describe more complex techniques in the Methods section.*
☒	☐	A description of all covariates tested
☒	☐	A description of any assumptions or corrections, such as tests of normality and adjustment for multiple comparisons
☐	☒	A full description of the statistical parameters including central tendency (e.g. means) or other basic estimates (e.g. regression coefficient) AND variation (e.g. standard deviation) or associated estimates of uncertainty (e.g. confidence intervals)
☒	☐	For null hypothesis testing, the test statistic (e.g. *F*, *t*, *r*) with confidence intervals, effect sizes, degrees of freedom and *P* value noted<br>*Give P values as exact values whenever suitable.*
☒	☐	For Bayesian analysis, information on the choice of priors and Markov chain Monte Carlo settings
☒	☐	For hierarchical and complex designs, identification of the appropriate level for tests and full reporting of outcomes
☒	☐	Estimates of effect sizes (e.g. Cohen's *d*, Pearson's *r*), indicating how they were calculated

Our web collection on statistics for biologists contains articles on many of the points above.

Software and code

Policy information about availability of computer code

Data collection	Micro-Manager (1.4 and 2.0), Fiji ACCeNT plugin (v1.0-beta), and custom software available at https://github.com/jdeschamps/htSMLM
Data analysis	Matlab (2020a), ImageJ (v1.53n), Fiji ACCeNT plugin, and custom software available at https://github.com/jries/SMAP. NCS code run in MATLAB 2020a (Supplementary Software of the original publication downloaded from nature.com). ACsN code run in MATLAB 2020a (Supplementary Software of the original publication downloaded from nature.com)

For manuscripts utilizing custom algorithms or software that are central to the research but not yet described in published literature, software must be made available to editors and reviewers. We strongly encourage code deposition in a community repository (e.g. GitHub). See the Nature Portfolio guidelines for submitting code & software for further information.

Data

Policy information about availability of data

All manuscripts must include a data availability statement. This statement should provide the following information, where applicable:

- Accession codes, unique identifiers, or web links for publicly available datasets
- A description of any restrictions on data availability
- For clinical datasets or third party data, please ensure that the statement adheres to our policy

Example data can be downloaded from https://rieslab.de/#accent with no restriction in access. Additional data supporting the findings of this study are available from the corresponding author, Jonas Ries, upon request.

Field-specific reporting

Please select the one below that is the best fit for your research. If you are not sure, read the appropriate sections before making your selection.

- [x] Life sciences
- [ ] Behavioural & social sciences
- [ ] Ecological, evolutionary & environmental sciences

For a reference copy of the document with all sections, see nature.com/documents/nr-reporting-summary-flat.pdf

Life sciences study design

All studies must disclose on these points even when the disclosure is negative.

Sample size	No sample-size calculation was performed. The manuscript reports a new method of a camera characterization and presents no comparison between different biological conditions. This is not a life science study with comparative analyses of a certain sample size.
Data exclusions	Only data from experiments that obviously failed (wrong microscope setup/out-of-focus) were excluded. Exclusion criteria were predetermined.
Replication	Replicability of camera calibration was proven and is shown in Supplementary Figure 2.
Randomization	No randomization was performed. This is not a life science study with comparative analyses of biological situations.
Blinding	No blinding was performed. There is no comparison of different biological situations performed in this work.

Reporting for specific materials, systems and methods

We require information from authors about some types of materials, experimental systems and methods used in many studies. Here, indicate whether each material, system or method listed is relevant to your study. If you are not sure if a list item applies to your research, read the appropriate section before selecting a response.

Materials & experimental systems

n/a	Involved in the study	
☐	☒	Antibodies
☐	☒	Eukaryotic cell lines
☒	☐	Palaeontology and archaeology
☒	☐	Animals and other organisms
☒	☐	Human research participants
☒	☐	Clinical data
☒	☐	Dual use research of concern

Methods

n/a	Involved in the study	
☒	☐	ChIP-seq
☒	☐	Flow cytometry
☒	☐	MRI-based neuroimaging

Antibodies

Antibodies used	Anti beta-tubulin antibody, catalog no. T5293, Sigma-Aldrich<br>Anti GFP antibody, catalog no. 598, MBL International,<br>Anti-rabbit secondary i1 DNA-PAINT antibodies, homemade, kind gift of Ingmar Schoen, Royal College of Surgeons in Ireland.
Validation	Validations were performed by the respectively indicated manufacturers/provider. These antibodies were used to create specimens for demonstrating the described camera characterization method. The specificity in immunostaining serves as an internal validation.

Eukaryotic cell lines

Policy information about cell lines

Cell line source(s)	U2OS Nup96-SNAP-tag cells (catalog no. 300444), U2OS Nup96-mEGFP cells (catalog no. 300174), U2OS Nup107-SNAP-tag cells (catalog no. 300294), are from CLS Cell Line Service, Eppelheim, Germany.<br>U373 cells expressing AP2-eGFP generously provided by the Boulant lab, German Cancer Research Center (DKFZ), Heidelberg, Germany
Authentication	Cell lines were not further authenticated.
Mycoplasma contamination	Cells were tested negative for mycoplasma contamination.
Commonly misidentified lines (See ICLAC register)	No commonly misidentified cell lines were used.
